# Comparative Growth Inhibition of Bread Spoilage Fungi by Different Preservative Concentrations Using a Rapid Turbidimetric Assay System

**DOI:** 10.3389/fmicb.2021.678406

**Published:** 2021-06-08

**Authors:** Marcelo Valle Garcia, Esther Garcia-Cela, Naresh Magan, Marina Venturini Copetti, Angel Medina

**Affiliations:** ^1^Department of Technology and Food Science, Center of Rural Sciences, Federal University of Santa Maria – UFSM, Santa Maria, Brazil; ^2^Applied Mycology Group, Cranfield Soil and Agrifood Institute, Cranfield University, Cranfield, United Kingdom; ^3^Biological and Environmental Sciences, School of Life and Medical Sciences, University of Hertfordshire, Hatfield, United Kingdom

**Keywords:** abiotic factors, spoilage fungi, turbidimetric assay, growth inhibition, time to detection

## Abstract

Bread and intermediate moisture bakery products are mainly spoiled by yeasts and filamentous fungi. The inoculum load and preservation system used determines their shelf life. To extend the shelf life of such commodities, the use of chemical preservatives is the most common way to try and control the initiation of mold spoilage of bread. This study has utilized a rapid turbidimetric assay system (Bioscreen C) to examine the temporal efficacy of calcium propionate (CP) and potassium sorbate (PS) for controlling the growth of important bread spoilage fungi. The objectives were to compare the temporal growth of strains of three important spoilage fungi *Hyphopichia burtonii* (HB17), *Paecilomyces variotii* (PV11), and *Penicillium roqueforti* (PR06) isolated from visibly molded bread to (a) different concentrations of CP and PS (0–128 mM), (b) temperatures (25°C, 30°C), (c) water activity (a_w_; 0.95, 0.97), and (d) pH (5.0, 5.5). All three abiotic factors, pH, a_w,_ and temperature, and preservative concentrations influenced the relative growth of the species examined. In general, PS was more effective than CP in inhibiting the growth of the strains of these three species. In addition, the Time to Detection (TTD) for the efficacy of the preservatives under the interacting abiotic factors was compared. The strain of *Paecilomyces variotii* (PV10) was the most tolerant to the preservatives, with the shortest TTD values for both preservatives. *P. roqueforti* was the most sensitive with the longest TTD values under all conditions examined. These results are discussed in the context of the evolution of resistance to food-grade preservatives by such spoilage fungi in bakery products.

## Introduction

Bakery products are intermediate moisture foods (approx. 0.95 a_w_; pH 5.0–6.0) and are thus prone to mold spoilage without the addition of control strategies, such as food-grade preservatives, modified atmosphere packaging, and storage, and the use of humectants (Magan and Aldred, 2006; Pitt and Hocking, 2009; Dagnas and Membré, 2013; Rodríguez et al., 2016; Garcia et al., 2019a). These are combined to develop effective shelf life for products that are stored under ambient conditions (e.g., bread products, cakes). However, with consumer pressure to reduce the use of preservatives, alternative natural preservatives are now being sought. However, some spoilage fungi have evolved to become more tolerant of the existing preservatives used (Suhr and Nielsen, 2004). Thus, more accurate data are required on the relative sensitivity/resistance of important spoilage molds to preservatives used in such leavened baked goods.

It is difficult to obtain estimates of the losses in the bakery product industry attributed to fungal spoilage and thus product wastage. This is influenced by season, product formulation and processing methods, and the hygiene status of the production and processing areas (Legan, 1993; Magan and Aldred, 2006). Although updated data were not available, Killian and Krueger (1983) estimated losses of around 5% in the USA and 1–5% in Europe (Abellana et al., 1997), while Freire (2011) showed that losses could exceed 10% in Brazil.

The genera *Penicillium* (*Penicillium roqueforti*, *Penicillium brevicompactum*, and *Penicillium chrysogenum*), *Wallemia*, some *Aspergillus glaucus* group (formerly *Eurotium*), and other common molds, including *Chrysonilia sitophila*, *Rhizopus* sp., *Mucor* sp., *Hyphopichia burtonii* (chalky mold), and *Paecilomyces variotii*, are amongst the species that most commonly have been shown to be involved in spoilage of bread (Pitt and Hocking, 2009; Alcano et al., 2016; dos Santos et al., 2016; Garcia et al., 2019). During the bread-making process, most of these are usually controlled using chemical preservatives, predominantly based on organic acids and their salts and include sorbate, benzoate, and propionate (Gioia et al., 2017). These are especially effective at lowered pH values (pH 4.0–5.5). Most of these preservatives are generally recognized as safe (GRAS) by the Food and Drug Administration (FDA), which generally means that they can be employed in the control of microbial contamination in raw ingredients and finished food products (Garcia-Garcia and Searle, 2016). However, several reports have indicated the existence of tolerance to these preservatives. For example, strains of *P. roqueforti* were found to be resistant to the recommended concentrations of calcium propionate (CP; 3,000 ppm) used in bread and were found to grow in treated bread matrices (Harris et al., 1986; Suhr and Nielsen, 2004). In addition, it was shown that, because these aliphatic acids and their salts are fungistatic, not fungicidal, the use of less than the recommended concentrations may facilitate growth and indeed mycotoxin production, which may have implications for food safety and consumer health (Schmidt-Heydt et al., 2007, 2013; Alcano et al., 2016).

Medina et al. (2012) showed that the Bioscreen C system, which has been previously used for bacteria and yeast studies, could also be used for filamentous growth assays by subtle modifications of the liquid broth medium. This has allowed the rapid screening of the efficacy of novel compounds, the ecology of growth and mycotoxin production by mycotoxigenic spoilage fungi, and the comparison of formulations of biocontrol agents (Medina et al., 2012; Aldars-García et al., 2018; Carbó et al., 2018; Debonne et al., 2020; García-Díaz et al., 2020).

The objectives of this study were to examine the relative sensitivity/tolerance of strains of three different fungal species isolated from visibly spoiled bread [*H. burtonii* (HB17), *P. variotii* (PV10), and *P. roqueforti* (PR06)] on the efficacy of a range of concentrations (0–128 mM) of CP and potassium sorbate (PS) on their temporal growth using the Bioscreen C assay system. This facilitated the comparison of the efficacy of these two preservatives on (a) relative growth inhibition in relation to interacting abiotic factors of temperature (25 and 30°C), water activity (a_w_, 0.95, 0.97), and pH (5.0, 5.5) and (b) the use of the relative Time To Detection (TTD) values to identify which of these three species may be more sensitive/resilient to these two food-grade preservatives.

## Materials and Methods

### Fungal Strains, Culture Media Preparation, and Preparation of Inocula

For this study, one strain of three different species isolated from visibly spoiled commercial bread was used. These strains were: *H. burtonii* (HB17), *P. variotii* (PV11), and *P. roqueforti* (PR06). They were isolated from sliced bread produced in a medium-sized production facility in Brazil (Latitude: 29° 41′ 03″ S; Longitude: 53° 48′ 25″ W). The full species identification procedure is described in Garcia and Copetti (2019). Only one representative strain of each species was selected for the present study as no statistical difference was found in previous assays carried out with multiple strains of each fungal species isolated from molded bread (Moro, 2018).

The *P. roqueforti* strain was sub-cultured on Malt Extract Agar (MEA) [20 g malt extract (Difco), 2 g peptone (Difco), 15 g agar (Sigma Aldrich, Dorset, UK)] for 7 days at 25°C in the dark, while *H. burtonii* and *P. variotii* strains were cultured in the same medium, but at 30°C, for 7 days (Pitt and Hocking, 2009).

The fungal strains grown on MEA were used to prepare a spore suspension adjusted to 10^5^ cells/spores/ml in sterile water +0.05% Tween 80 solution. The initial concentrations were determined with a Thoma hemocytometer (Marienfeld, Lauda-Königshofen, Germany) and then diluted using sterile distilled water.

### Preservatives

The main preservatives (salts of the two aliphatic acids) employed by the bread industry were used to conduct this study. CP (C_6_H_10_CaO_4_; Sigma Aldrich, Dorset, UK, >99%), was used in the following concentrations 0 (control), 2, 4, 8, 16, 32, 64, and 128 mM (=0; 372; 745; 1,490; 2,979; 5,959; 11,918; and 23,836 ppm respectively). PS (C_6_H_7_KO_2_; Sigma Aldrich, Dorset, UK, >99%) was used in the same concentrations as CP in mM, and this corresponded to 0; 300; 601; 1,202; 2,403; 4,807; 9,614; and 19,228 ppm, respectively. The different concentrations of each preservative were added to Yeast Extract Sucrose (YES) broth + 0.05% agar (Technical agar No. 2) medium with pre-determined pH (5.0 or 5.5) and a_w_ (0.95 or 0.97) values (Medina et al., 2012). The pH and a_w_ values used in these studies were chosen based on the intrinsic parameters of industrial bread marketed in Brazil. The pH ranged from 5.18 to 5.49 according to Moro (2018), and the a_w_ from pan bread on the first day ranged from 0.94 to 0.98 (Ishida and Steel, 2014). Also, the temperatures chosen were based on the storage temperature of bakery products in tropical countries in supermarkets and by consumers. Some Brazilian and international regulations were considered for the determination of the tested doses (Brazil, 2010; EU, 2011), including the established recommended values for the control of fungal growth at different pH values.

### Turbidimetric Assays

For the Bioscreen C assays, YES semi-solid broth medium (400 ml) containing 20 g of yeast extract, 150 g of sucrose, 0.5 g magnesium sulfate, and 0.125% of technical agar (Sigma Aldrich Ltd), was used, as recommended by Medina et al. (2012). Acetate buffer at pH 5.0 and 5.5 instead of water was used for the adjustment of the medium pH. Glycerol/water mixtures were used to make up the buffers at the target water activity levels (a_w_; 0.95 and 0.97).

The efficacy of different concentrations of the preservatives on temporal growth was quantified using three Bioscreen C Microbiological Growth Analyzers (Labsystems, Helsinki, Finland). The 100-well microtiter plates were loaded with 200 μl of the semi-solid YES media with preservative treatment conditions (pH; a_w_) and inoculated with spore suspensions of each strain. The temporal change in the optical density (OD) was recorded every 20 min using the 600 nm filter for 6 days (8,640 min.) since it is directly related to the changes in fungal biomass (Medina et al., 2012).

Data were recorded using the software Easy Bioscreen Experiment (EZExperiment) provided by the manufacturer and then exported to a Microsoft Excel Professional 2010 (14.0.4756.1000; Microsoft Corporation, Redmond, Washington, United States) sheet for further analysis. Experiments were conducted at 25 and 30°C, with five replicates per treatment and the experiments were repeated once.

### Comparative Temporal Growth of the Species in Different Concentrations of the Preservatives

The temporal growth patterns up to the final reading in each replicate well up to 8,640 min (6 days) was measured, and these were compared to visualize the efficacy of the different preservative concentrations on each species. In addition, the final absorbance values of each treatment were plotted to compare the relative inhibition.

### Time to Detection of Fungal Strains in Relation to Preservative Concentrations × Abiotic Factors

The datasets obtained from the Bioscreen C were subjected to further analysis. The TTD for an OD of 0.2 (for *P. variotii* and *P. roqueforti*) and an OD of 0.4 (for *H. burtonii*) was obtained using a Microsoft® Excel® template (kindly provided by Dr. R.J.W. Lambert), which used linear interpolation between successive OD readings. These TTD OD values were chosen because they more accurately represented the initiation of growth before the filamentous development for these fungi (Carbó et al., 2018; García-Díaz et al., 2020).

### Data Analysis

Once the final OD and TTDs were obtained, ANOVA was performed by comparing the final OD or TTD among the same fungal strain in all the different treatments and preservatives. Furthermore, multiple mean comparisons were performed using the Scott-Knott test (*p* < 0.05), and the SISVAR® Software version 5.6 (DEX, UFLA, Brazil) was used in the analysis.

## Results

### Comparative Inhibition of Growth of the Three Species by the Preservatives

**Figure 1:**
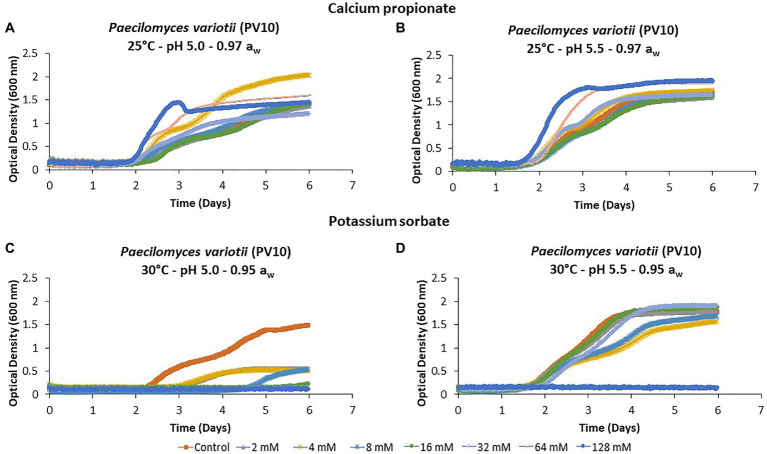
Growth curves of *Paecilomyces variotii* (PV10) in **(A,B)** in the presence of calcium propionate (CP), and **(C,D)** potassium sorbate (PS) in semi-solid Yeast Extract Sucrose (YES) media containing different concentrations (0–128 mM) over 6 days incubation using the BioScreen **(C)**. Each curve represents the mean of five replicates.

Figure 1 provides an example of the mean temporal growth curves of five replicates of *P. variotii* (PV10) obtained after inoculation with different concentrations of CP and PS at the different pH × temperatures × a_w_ conditions tested over 6 days. CP was not very effective at controlling the growth of *P. variotii* in any of the conditions tested. Indeed, at some concentrations, growth was slightly enhanced (Figures 1A,B). In contrast, PS had better efficacy with a reduction in temporal growth by >66% at pH 5.0 after 6 days (Figure 1C). The inhibitory effect was reduced at pH 5.5, where less of the increasing concentrations significantly reduced fungal growth, especially at 0.95 a_w_ (Figure 1D).

**Figure 2:**
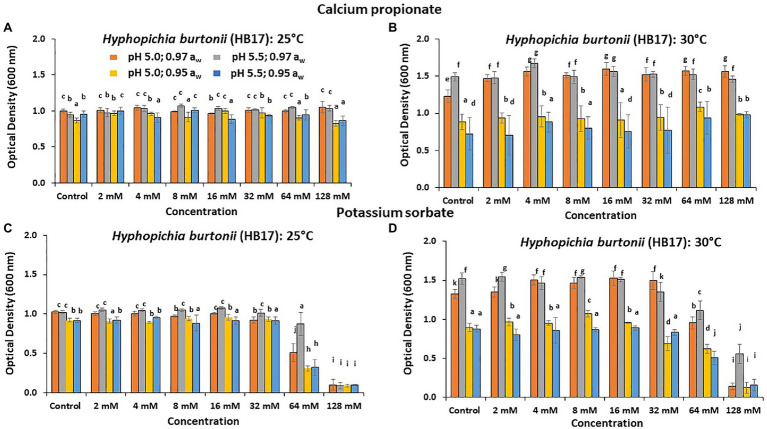
Effect of CP on growth (8,640 min, 6 days) of *Hyphopichia burtonii* (HB17) at **(A)** 25°C and **(B)** 30°C. The effect of PS on growth is shown in **(C)** 25°C and **(D)** 30°C, respectively. Bars represent the SEM. Treatments with different letters are significant (*p* = 0.05).

Figure 2 shows the effect of CP and PS on the relative inhibition of the growth of *H. burtonii* after 6 days. PS had a significant effect in controlling (*p* < 0.05) the growth of *H. burtonii* (HB17). For CP, at 25°C/pH 5.0 and 0.97 a_w_ this spoilage yeast was more resilient and was able to grow effectively when compared to other treatments at the same concentration. After 6 days, the highest concentration tested had good efficacy at 0.95 a_w_, (OD 0.83 and 0.86, *p* < 0.05) and 25°C. For PS, a significant (*p* < 0.05) inhibitory effect was observed at 25 and 30°C, with the highest concentrations examined (64 and 128 mM) in the 0.95 a_w_ treatments (see Figures 1C,D). At 30°C, the highest concentration (128 mM) was required for effective inhibition.

Supplementary Figures S1A,B provide additional information on the comparison between the controls and 32–128 mM of both preservatives for *P. variotii* and *P. roqueforti* after 6 days. These again show the influence of the temperature x pH x a_w_ on the relative effects of the two preservatives on the growth of these species.

### Effect of Preservatives on the Time to Detection for the Growth of the Different Fungal Species

**Figure 3:**
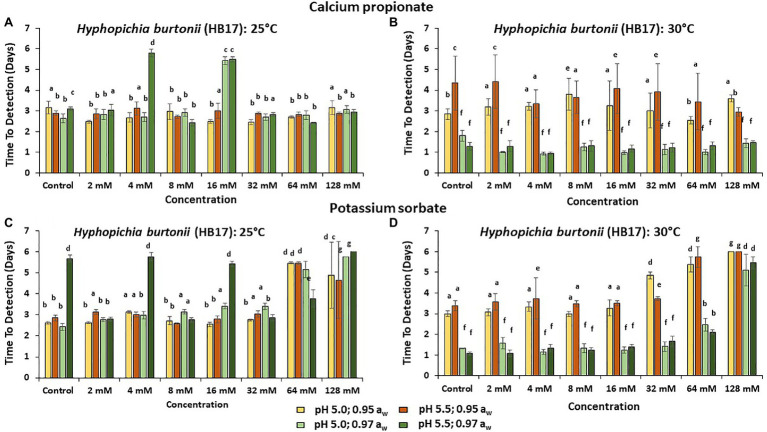
The Time to Detection (TTD; OD = 0.4 OD; 6 days) of HB17 at **(A)** 25°C and **(B)** at 30°C in the presence of different concentrations of CP; and for PS at **(C)** 25°C and **(D)** 30°C. Bars represent the SEM. Treatments with different letters are significant (*p* = 0.05).

**Figure 4:**
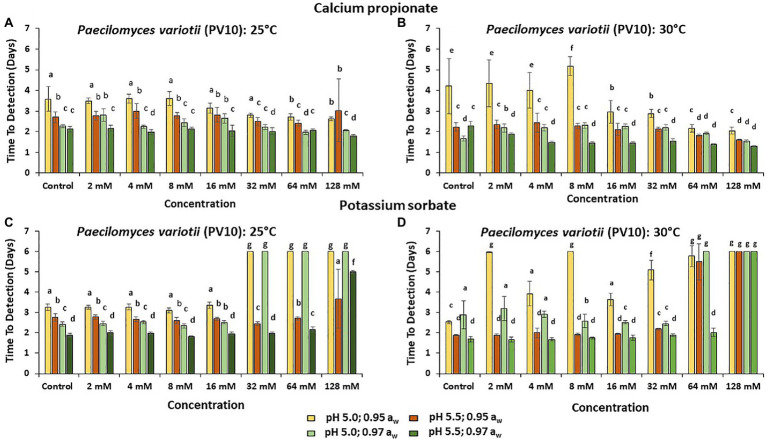
The TTD (OD = 0.4 OD; 6 days) of *P. variotii* (PV10) at **(A)** 25°C and **(B)** at 30°C in the presence of different concentrations of CP; and for PS at **(C)** 25°C and **(D)** 30°C. Bars represent the SEM. Treatments with different letters are significant (*p* = 0.05).

**Figure 5:**
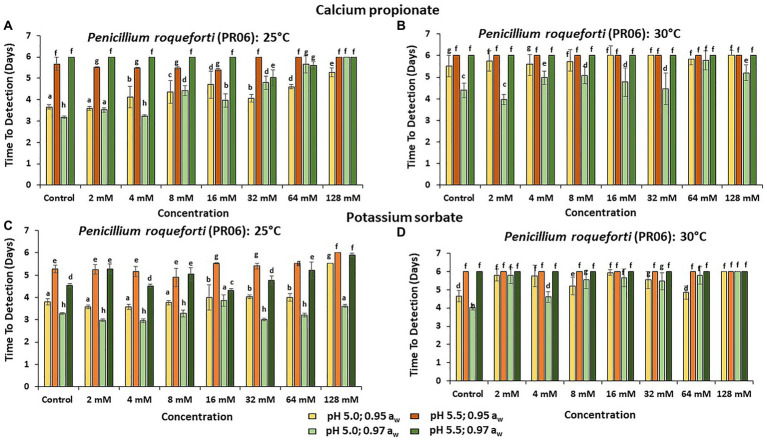
The TTD (OD = 0.4 OD; 6 days) of *Penicillium roqueforti* (PR06) at **(A)** 25°C and **(B)** at 30°C in the presence of different concentrations of CP; and for PS at **(C)** 25°C and **(D)** 30°C. Bars represent the SEM. Treatments with different letters are significant (*p* = 0.05).

Figures 3–5 show the TTD means for the strains of these three species examined under all the conditions tested. Overall, PS showed the best inhibition values with the longest TTD values (*p* < 0.05) obtained, especially when exposed to 64 and 128 mM concentrations when compared to CP.

In the presence of CP at 25°C, *H. burtonii* (HB17) had the longest TTD periods. These were >5.0 days at 4 and 16 mM concentrations in the 0.97 a_w_ treatments (Figure 3A). At 30°C, the longest TTD values (*p* < 0.05) were obtained at 0.95 a_w_ (Figure 3B). For PS, the highest preservative concentration tested (128 mM) was required for effective control of growth 30°C (Figure 3D). The a_w_ reduction positively reduced the initiation of growth of *H. burtonii* at 30°C in all cases (Figures 3B,D), showing a higher inhibition when compared to the preservative effect itself in the control.

The strain of *P. variotii* (PV10) was tolerant to all CP concentrations at 25°C. Indeed, at 30°C, increasing the concentrations of CP appeared to stimulate growth (Figures 4A,B). However, this strain was unable to initiate growth at pH 5.0 and >32 mM of PS (*p* < 0.05). For *P. variotii*, increasing the temperature to 30°C resulted in better inhibition by PS, mainly at pH 5.0, with no growth initiated during the time frame of the experiments (6 days; Figures 4C,D).

The strain showing the longest TTD periods was *P. roqueforti* (PR06), both in the presence or absence of preservatives, especially when the higher pH value is examined (Figure 5).

## Discussion

Fungal spoilage of bread is an ongoing problem in the food industries requiring the use of preservatives for obtaining the necessary shelf life for such products under ambient storage conditions. The present study has isolated these strains directly from spoiled bread in a production facility (Garcia et al., 2019a). Among the different fungal species isolated, *H. burtonii* was commonly found, as it is known to be responsible for causing the “chalk-moldy” defects on bread (Deschuyffeleer et al., 2011; Garcia et al., 2019a). *P. variotii* and *P. roqueforti* were also often found in bakery production plants with poor hygiene.

This is the first detailed study using the BioScreen C rapid bioassay system for the examination of the effect of commonly used preservatives in the bread/bakery product production process and of the possible differential effects of preservatives on the control of such spoilage fungi. The present study has shown that it was possible to obtain consistent and reproducible results using this system to compare the efficacy of these two commonly used preservatives on relative control of growth under different interacting abiotic conditions simulating conditions/preservatives in leavened bread products. In general, PS was more effective than CP in controlling the growth of *H. burtonii* and *P. variotii*, but it was not clear for *P. roqueforti*. In addition, this could be a valuable rapid alternative tool for alternatives to challenge testing to examine the shelf life of newly formulated bakery products when using different preservatives (Lambert and Pearson, 2000; Lambert and Lambert, 2003).

The concentration of CP allowed by Brazilian legislation is considered *quantum satis*, while that allowed by EU has a maximum in the range of 1,000–3,000 ppm. For PS, the maximum allowable concentration is 2,000 ppm in the EU, and 1,000 ppm in Brazil (Brazil, 1999, 2010; EU, 2011). The range of concentrations used in the present study included a number of doses above the recommended maximum concentrations of salts of these aliphatic acids in bakery products (≥32 mM). However, it should be noted that the strains of all three species were able to grow in the presence of 64 and 128 mM, which represents >2–4 times higher concentrations than that recommended in bread and bakery products. Of course, these preservatives are fungistatic, not fungicidal, and thus the inoculum present remains viable, although growth may be inhibited.

The resilience in the presence of these preservatives was demonstrated by the strain of *H. burtonii*. This was tolerant to relatively high concentrations of both CP and PS. Previously, Magan and Lacey (1986) found a strain of this yeast in an animal feed treated with >100 mM of ammonium propionate, and this strain was able to rapidly metabolize this preservative as a carbon source. Other studies have shown strains of *H. burtonii* to also be tolerant of 100 mM sodium propionate (Moon, 1983). The present study confirmed the resilience of strains of this spoilage yeast in a range of commodities destined for food or feed use. Previous studies by Stratford et al. (2009, 2013) also suggested that perhaps different mechanisms of action of preservatives, such as acetic and sorbic acid, against other spoilage yeasts, such as *Zygosaccharomyces bailii*, may occur, which cannot be explained by the traditional weak-acid theory. A recent study on spoilage yeasts in low sugar beverages suggested that sorbic acid inhibited the respiration activity of spoilage yeasts more strongly than fermentation (Stratford et al., 2020). This may strengthen the preservative efficacy of weak acids against spoilage yeasts because of this effect.

The relative sensitivity of *P. roqueforti* to the preservatives might be related to the characteristics of this species and to the conditions utilized in the present study. *P. roqueforti* is a psychrotrophic fungus, with maximum growth temperature near above 30°C, and when other conditions were further away from the optima (Frisvad and Samson, 2004; higher pH and lowered a_w_), growth initiation was completely inhibited for the experimental period (6 days). It is also possible that the acetate buffer used in the present study may have influenced the growth of *P. roqueforti*. A previous study compared the growth of *P. roqueforti* to that of *Penicillium verrucosum* under different conditions (Meyers and Knight, 1958; Veau et al., 1981; Stiles et al., 2002). They used a medium containing sodium acetate at 58 g/L to differentiate these two species. The growth of the *P. roqueforti* strain was completely inhibited. The present study showed that the TTD periods for *P. roqueforti* were longer at pH 5.5, where the highest concentration of acetate buffer was present. Indeed, acetate formation by heterofermentative lactic acid bacteria in sourdough bread was found to delay the growth of *P. roqueforti* due to increased acetate formation, thus prolonging the shelf life of bread (Quattrini et al., 2019). Similarly, Fredlund et al. (2004) observed that the ethyl acetate produced by *Pichia anomala* in glass tubes was responsible for the inhibition of *P. roqueforti* growth at 0.95 and 0.98 a_w_.

Previous studies of *P. roqueforti* strains have found them to be relatively tolerant of some food-grade preservatives. For example, *P. roqueforti* was found to be tolerant of both CP and sodium benzoate (SB; Tzatzarakis et al., 2000; Marin et al., 2002; Suhr and Nielsen, 2004; Guynot et al., 2005). Strains from sourdough bread were found to be tolerant of CP at >3,000 ppm with germination of spores and colonization occurring during storage of the bread (Harris et al., 1986; Suhr and Nielsen, 2004). Indeed, Suhr and Nielsen (2004) also showed that higher CP concentrations (0.3%) at 0.97 a_w_ stimulated *P. roqueforti* growth.

The results for *P. variotii* showed that both CP and PS were more effective at pH 5.0 than 5.5 and 0.95 than 0.97 a_w_. There is less information available on the effect of preservatives on this spoilage species. Studies by Lord et al. (1981a) showed that *P. variotii* and *A. glaucus* were able to grow in the presence of up to 135 mM of propionate in the hay. These studies also showed the importance of effective mixing of the preservatives with the matrix because under-treated pockets could allow initiation of growth, even if this was only 1–2 gm of untreated pockets because of poor mixing. They also showed that mixtures of salts of aliphatic acids may be better, although some of them could be utilized as carbon sources (Lord et al., 1981b; Lacey et al., 1983). This again emphasizes the importance of using the right concentration of the preservative and the importance of homogeneous integration into the bakery product during production.

Studies of decimal reductions of the EU recommended concentrations of CP and PS (3,000 vs. 300 ppm) and effects on *P. verrucosum* growth and production of the mycotoxin ochratoxin A (OTA) was examined in bread analogues under interacting conditions of pH × a_w_ at 25°C (Arroyo et al., 2005). This showed that efficacy was best with the recommended concentrations, especially at pH 4.5 and 0.93–0.95 a_w_. However, with lower concentrations of these preservatives, there was a stimulation of growth and an increase in OTA production, especially at pH 6 and 0.93–0.95 a_w_. This was supported by more detailed studies by Schmidt-Heydt et al. (2007) who showed that both growth and OTA production by *P. verrucosum* was stimulated by decimal reductions of these preservatives. This was supported by the relative increase in expression of the *otapks*PV gene, which is important in the biosynthetic pathway of OTA production.

This certainly suggests that the risks of spoilage initiation and indeed mycotoxin contamination could be increased with reduced concentrations of such preservatives.

This study has shown that the strains of *H. burtonii* and *P. variotii* have developed very good resilience to the existing preservatives, even at concentrations much higher than those allowed in the EU and many other countries. Generally, efficacy was better at pH 5.0 and 0.95 a_w_ when compared to that at pH 5.5. and 0.97 a_w_. Of course, the efficacy of the preservatives is normally much better at their pKa values when 50% dissociation occurs. These values are closer to ≤pH 5 than to ≥pH 5.5. In contrast, the *P. roqueforti* strain appeared to be more sensitive to the range of concentrations used with very good inhibition based on the TTD periods. It is possible that the former strains under the conditions and use of preservatives in bakery product plants have evolved to develop some resilience to the existing preservatives, perhaps as there are no strict guidelines on maximum concentrations in bakery commodities in Brazil. Overall, more diverse strains need to be tested to examine whether this is a consistent trend or whether there is significant interstrain variation in terms of sensitivity/tolerance to these preservatives under interacting abiotic conditions.

## Conclusion

There were some differences in the behavior of the strains of the three species isolated from bread in Brazil. In general, the inhibition of growth and the TTD periods showed that PS was more effective than CP. *P. variotii* (PV10) was the most resistant strain, regardless of the pH × a_w_ × temperature used in these studies. In contrast, the strain of *P. roqueforti* was the most sensitive with the longest TTD periods, indicating the effectiveness of both preservatives. Studies are now needed on more naturally isolated strains of these species from bakery product production plants to understand the evolution of potential resistance to these preservatives and to determine whether alternatives may be required to obtain the necessary shelf life of such products and reduce food waste.

## Data Availability Statement

The raw data supporting the conclusions of this article will be made available by the authors, without undue reservation.

## Author Contributions

MG carried out the research work and data analyses. EG-C helped with rapid bioassays, data analyses, data interpretation and writing. MC was the primary research supervisor in Brazil and manuscript drafting. NM was co-supervisor of research at Cranfield and manuscript writing. AM was primary research supervisor at Cranfield and manuscript writing. All authors contributed to the article and approved the submitted version.

### Conflict of Interest

The authors declare that the research was conducted in the absence of any commercial or financial relationships that could be construed as a potential conflict of interest.

## Supplementary Material

Supplementary Figure S1 **(A)** Effect of Calcium propionate (CP) and Potassium sorbate (PS; 0; 32–128 mM) on relative growth (6 days) for *Paecilomyces variotii* (PV10) at 25°C and 30°C; **(B)** effect of these two preservatives on the relative growth of *Penicillium roqueforti* (PRO6).
