# Supplementary figures and images for: Comparative Growth Inhibition of Bread Spoilage Fungi by Different Preservative Concentrations Using a Rapid Turbidimetric Assay System
Source: Front Microbiol. 2021 Jun 8;12:678406. doi: 10.3389/fmicb.2021.678406 (PMC8219074; doi:10.3389/fmicb.2021.678406)

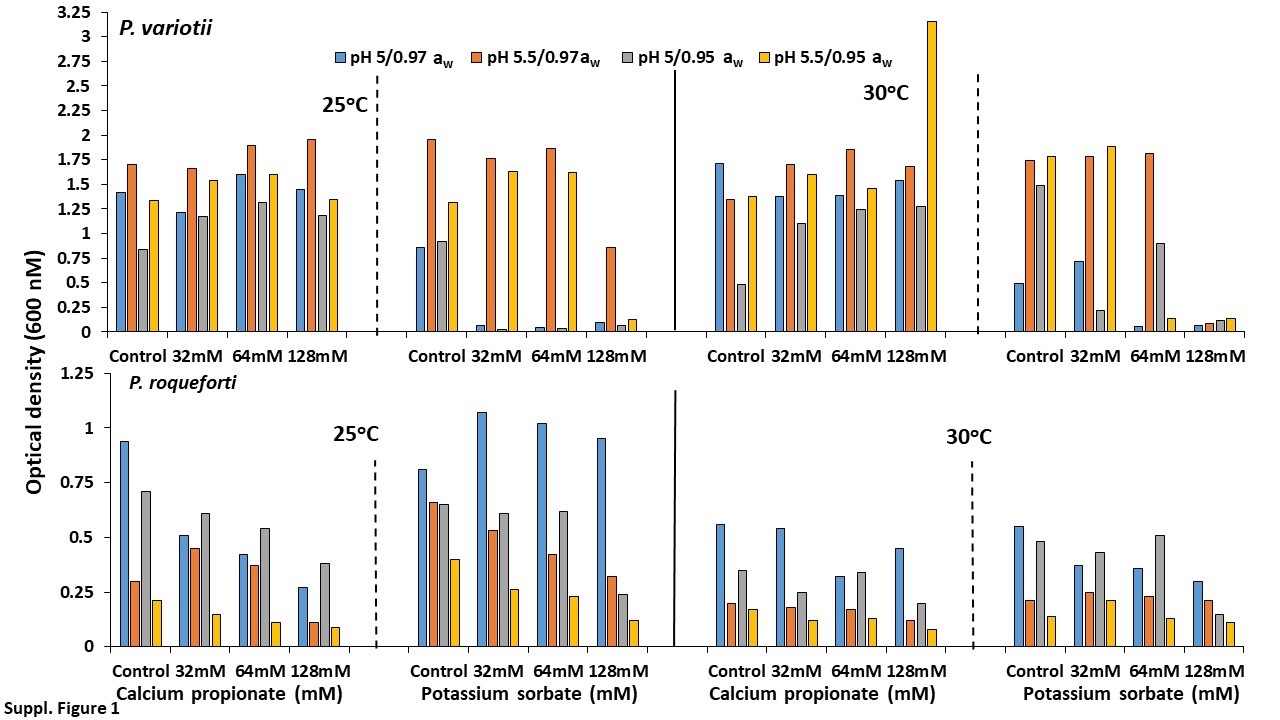

Supplement: Supplementary Figure S1 — (A) Effect of Calcium propionate (CP) and Potassium sorbate (PS; 0; 32–128 mM) on relative growth (6 days) for Paecilomyces variotii (PV10) at 25°C and 30°C; (B) effect of these two preservatives on the relative growth of Penicillium roqueforti (PRO6). [file Image_1.JPEG]
